# Sensory Metabolite Profiling in a Date Pit Based Coffee Substitute and in Response to Roasting as Analyzed via Mass Spectrometry Based Metabolomics

**DOI:** 10.3390/molecules24183377

**Published:** 2019-09-17

**Authors:** Mohamed A. Farag, Asmaa M. Otify, Aly M. El-Sayed, Camilia G. Michel, Shaimaa A. ElShebiney, Anja Ehrlich, Ludger A. Wessjohann

**Affiliations:** 1 Pharmacognosy Department, Faculty of Pharmacy, Cairo University, Cairo 11562, Egypt; 2 Department of Chemistry, School of Sciences and Engineering, The American University in Cairo, New Cairo 11835, Egypt; 3 Toxicology and Narcotics Department, National Research Centre, Giza 12622, Egypt; 4 Leibniz Institute of Plant Biochemistry, Dept. Bioorganic Chemistry, Weinberg 3, D-06120 Halle (Saale), Germany

**Keywords:** *Phoenix dactylifera*, date palm, coffee, volatiles, multivariate analysis

## Abstract

Interest in developing coffee substitutes is on the rise, to minimizing its health side effects. In the Middle East, date palm (*Phoenix dactylifera* L.) pits are often used as a coffee substitute post roasting. In this study, commercially-roasted date pit products, along with unroasted and home-prepared roasted date pits, were subjected to analyses for their metabolite composition, and neuropharmacological evaluation in mice. Headspace SPME-GCMS and GCMS post silylation were employed for characterizing its volatile and non-volatile metabolite profile. For comparison to roasted coffee, coffee product was also included. There is evidence that some commercial date pit products appear to contain undeclared additives. SPME headspace analysis revealed the abundance of furans, pyrans, terpenoids and sulfur compounds in roasted date pits, whereas pyrroles and caffeine were absent. GCMS-post silylation employed for primary metabolite profiling revealed fatty acids’ enrichment in roasted pits versus sugars’ abundance in coffee. Biological investigations affirmed that date pit showed safer margin than coffee from its LD_50_, albeit it exhibits no CNS stimulant properties. This study provides the first insight into the roasting impact on the date pit through its metabolome and its neuropharmacological aspects to rationalize its use as a coffee substitute.

## 1. Introduction

Food waste is considered as “one of the great paradoxes of our times”, with an increasing interest in the valorization of food products, utilization of its less used parts is urged as a necessity [1]. Food industries are interested in the economic utilization of food waste as valuable resources for other potential uses. Date pits are generally utilized as poultry and animal feed [2], encompassing high levels of dietary fibers which makes them suitable for preparing fiber based foods [3]. Moreover, date pit oil has proved to be an excellent biofuel source [3]; date pits are used as soil fertilizer [2]. An additional novel utilization includes roasting date pits for preparing a caffeine-free beverage to be used as coffee substitute. Famous coffee substitutes worldwide include dandelion, barley and malted barley [4].

In the Middle-East, particularly in the Arab world where the date palm grows as a major crop, date pit drink is used as a substitute for coffee beverages. Coffee-like preparations made from date pits are available in several Arab countries. Roasting date pits into a caffeine free drink could provide a substitute to satisfy consumer habitual coffee drinking while mitigating against caffeine related side effects (e.g., for hypertensive patients). 

Volatiles analysis of raw date pits revealed different classes of compounds, mainly alcohols, aldehydes, ketones, saturated and unsaturated hydrocarbons, terpenoids and esters [5]. Other than the date pit’s aroma, nothing is known about changes accompanying the roasting process needed to prepare it as coffee substitute. Some of the chemical and physical changes occurring during date pit roasting are likely to involve Maillard type and Strecker reactions, as occurs upon typical coffee seeds when roasting to incur most of the attributed changes in its sensory characteristics; viz., aroma or taste. Maillard reactions occur between reducing sugars and amino acids and/or proteins producing volatile pyrazines, pyrroles and pyridines. 

Headspace solid phase micro-extraction (SPME) is a relatively novel technique used for volatile analysis with the advantage of being solvent free and requiring no heat application, when compared to other methods. Moreover, SPME enables the enrichment of volatiles from gas or liquid samples, over a fused-silica fiber, followed by subsequent desorption of these analytes, thus leading to detection of less abundant volatiles as in roasted date pits [6]. We have applied such technology for the analysis of date fruit aroma from several varieties [6] and extend that herein to encompass that of its pit’s products.

No research has been performed on the determination of the quality characteristics of a coffee substitute beverage from roasted date pits (*Phoenix dactylifera* L.) compared to those of traditional coffee. The main goal of this study was to: (1) assess the metabolite changes that occur during date pit roasting; (2) identify key sensory chemicals of date pits and to distinguish them from those of coffee; and (3) determine any potential CNS effects for roasted date pit extracts in the context of the known effect from roasted coffee seeds.

## 2. Results and Discussion

To determine roasted date pit metabolite profiles, samples were subjected to detailed metabolomics analyses targeting its volatile and non-volatile metabolites, in comparison to those of unroasted pits and roasted coffee seeds. Data from each platform was further analyzed using multivariate data analyses to assist in identification of specimen’s markers or their classification in an untargeted manner.

### 2.1. Volatile Components and Their Contribution to the Aroma of Roasted Pits and Coffee via SPME Analysis

Aroma volatiles produced during coffee seeds’ roasting may be the most important determining factor of coffee quality and have been the subject of research interest for almost a century, with intensive profiling reports. Aroma analysis of roasted date pits was performed to identify how they differ from typical roasted coffee product. Roasted coffee’s aroma compounds are comprised of several chemical classes which are: thiols and other sulfur compounds, pyrroles, pyrans, pyrazines, furans, monoterpenoids and aromatics [7]. Quantitatively, the most abundant classes in coffee were to furans and pyrazines, whereas qualitatively, sulfur containing volatiles and pyrazines are regarded as the most significant contributors to coffee’s aroma [8]. These volatiles vary significantly among different coffee blends which makes flavor extremely complex, and explains why different coffee types may exhibit such diverse, unique and specific flavors. 

Date palm roasted pit as a coffee alternative is expected to exhibit a comparable, acceptable aroma and flavor considering its usage in the market. In this study, roasted date pit (RS) and two commercial products derived from roasted pit (P_1_ and P_2_) were analyzed via solid phase micro-extraction SPME-GC along with coffee product (C) to assess differences between roasted date pits and coffee seeds. 

**Figure 1:**
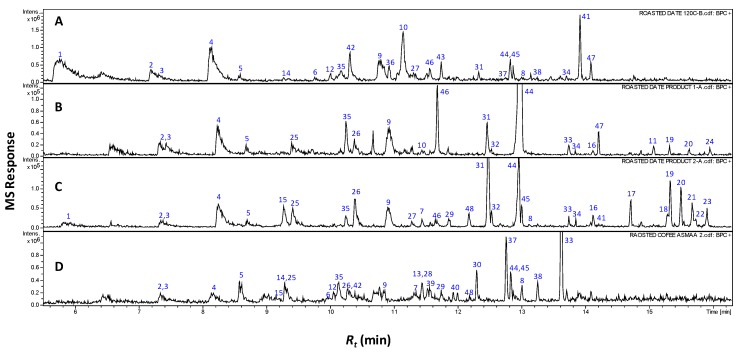
Representative SPME-GCMS chromatograms (*R_t_* 5–16 min) of headspace volatiles derived from roasted pits (**A**), pit product one (**B**), pit product two (**C**) and coffee product (**D**). The corresponding volatile names for each peak follow those listed in Table 1.

The complete the list of identified volatiles in home-prepared roasted dates (RS), commercial preparations (pit product two, P_2_) and those of coffee (C) samples are presented in Table 1, and a representative gas chromatogram is shown in Figure 1. It should be noted that raw date pits had no aroma and were thus not included in the SPME analysis. A total of 21 volatile compounds were detected in coffee specimens of which additionally four volatiles are identified for the first time in roasted coffee seeds.

This study represents, moreover, the first volatile characterization of roasted date pits, and roasting’s impact on their aroma. A total of 42 different volatile constituents were identified as key odorants in roasted date pits and their commercial products, with furans amounting to the major class, as is typical in coffee [9,10].

#### 2.1.1. Furans and Furanones

Furans are among the most abundant volatiles that characterize roasted coffee’s aroma, exhibiting a sweet, roasted smell, and are produced via thermal degradation of carbohydrates, ascorbic acid or unsaturated fatty acids upon roasting [9,10]. 

A total of nine furans and furanones were detected at high levels in all roasted pit samples. In RS and P_2_, they amount for 5.9%–26.5%, of their aromatic compounds, including 3-furfural (1), 2-acetyl-furan (2), dihydro-4-methyl-2(3H)-furanone (3), 5-methyl-2-furaldehyde (4), furfuryl acetate (5), 2-furfuryl furan (6), 5-methyl-2-furfuryl-furan (7), difurfuryl ether (8) and 2-furfuryl-methyl-disulfide (40) (Figure 1 and Table 1). Furanones are formed mainly via Maillard reaction and subsequent aldol condensation, imparting a sweet, caramelly aroma to roasted seeds in general [9,11]. All previous furans were also found in C samples with the sulfur containing furan (2-furfuryl-methyl-disulfide (40) though being detected for the first time in roasted coffee (Figure 1 and Table 1). The enrichment of furans in roasted date pit samples being similar to that of coffee justifies their similar characteristic, roasted, sweet aroma. 

#### 2.1.2. Pyrans

The group of pyran-4-ones is the most interesting one with respect to the sweet and burnt aroma characteristic [12]. Among pyrans, maltol (9) and 3-hydroxy-2,3-dihydro-maltol (10) were detected in all roasted pit and coffee specimens. Maltol imparts a caramel like smell to roasted coffee [13], and is derived from maltose degradation [14]. Upon over-roasting, coffee seeds increase in maltol levels [15] and whether such a scenario also occurs in date pits has yet to be reported. 

The presence of maltol and 3-hydroxy-2,3-dihydro-maltol in roasted pit samples at higher levels (8.6%–12.4%) compared to coffee (0.9%) suggests that they play an important role as key odorants of roasted date pits. It should be noted that pyrazines, reported as major components of roasted coffee volatiles, were not detected in roasted pit specimens. 

#### 2.1.3. Pyrroles

Although detected at similar levels in coffee samples as furans (5.7%), pyrroles are not considered potent odorants, owing to their high threshold values. As a group, furans impart burnt and caramel base notes while pyrroles express smoky and burnt coffee’s aromas, respectively [16]. Among the pyrroles reported for coffee, 2-acetyl-pyrrole (12) and 1-(2-furanyl-methyl)-1H-pyrrole were identified in our samples (13). However, lower levels of pyrroles were detected in the analyzed roasted pit specimens, reaching only 1% (RS), suggesting that their minor presence could distinguish the roasted pit aroma from that of coffee (Figure 1 and Table 1).

#### 2.1.4. Mono/Sesquiterpenoids

In contrast to coffee’s volatile blends that showed no sesquiterpenoids, such terpenoids amounted to major volatile components in commercially-roasted date products, amounting to 21.2% in P_2_, with *α*-copaene (16), (*E*,*β*)-farnesene (17), *α*-humulene (18), curcumene (19), *α*-zingiberene (20), *β*-bisabolene (21), *δ*-selinene (22), *β*-sesquiphellandrene (23) and calamenene (24) as the main components. In coffee specimens, monoterpenoids were present at only trace levels (0.1%) represented by *α*-phellandrene (15). The almost complete absence of such volatile class from home-prepared roasted date pits, and its exclusive presence in commercial products, suggests usage of additives in commercial pit products to enhance its flavor and overall quality, although the label does not inform consumers about these additional ingredients.

#### 2.1.5. Oxygenated Monoterpenoids

Oxygenated monoterpenoids contribute to green coffee seed aroma and are known to survive the roasting process [17]. Several oxygenated monoterpenes were identified in commercial pit products (up to 18.1% in P_2_), as well as coffee samples (45.6%) including cineole (25), linalool (26), *p*-menthone (27), isopulegone (28), *α*-terpineol (29), linalyl-acetate (30), pulegone (31), terpinyl-acetate (31), carvone (32) and eugenol (34) (Figure 1 and Table 1). The low level of oxygenated monoterpenes in home-prepared roasted date pits (1.2%) suggested that they might originate from additives added to improve the product’s sensory characters. This is the first report of cineole (25), linalyl acetate (30) and terpinyl acetate (31) in roasted coffee seeds, and their presence at such high levels (8.2%, 6.6% and 26.2%, respectively) suggests their inclusion as extra flavors in coffee.

#### 2.1.6. Phenolics

Phenolic compounds (guaiacol subclass) are commonly generated during the roasting of coffee seeds, and are thought to be potent odorants [10], among which 2-methoxy-phenol (35), 4-ethyl-guaiacol (37) and 4-vinyl-guaiacol (38) were identified in C samples at high levels (22.3%). They are typical of spicy and smoky aromas [11,18]. Although detected at relatively lower levels (0.3%–1.8%), such aromatic compounds were also identified in roasted pit samples. These phenolics are developed from thermal degradation of chlorogenic, quinic and other phenolic acids. 

#### 2.1.7. Sulfur Containing Volatiles

Sulfur containing volatiles are recognized as key contributors to the roasted flavor of coffee despite their presence at relatively low levels (0.4%), owing to their low smell threshold [19]. Thiophenes are possibly produced during roasting from sulfur containing amino-acids; i.e., cystine or methionine—known to also occur in green coffee seeds [20]. 2-Propionyl-thiophene (39) was identified in all samples typical of roasted and meaty flavor [12] in addition to 2-furfuryl-methyl-disulfide (40). A major sulfur containing volatile found in date pit samples (6.4%–42.3%) is benzyl-thiocyanate (41), known to exhibit a chemo-preventative effect aside from its pungent aroma, and is typically found in *Brassica* plants [21,22].

#### 2.1.8. Aldehydes/Ketones/Lactones

Aldehydes and ketones together account for most of the fruity smells in foods [23]. Nonanal (42), *n*-decanal (43) and 2-undecanone (45) were among the major aromatic constituents detected at 4.4% (RS), 1.6% (P_2_) and 0.5% in (C). In contrast, cyclotene or maple lactone (14) was detected at much lower levels in (RS) samples of a sweet caramel and spicy scent [24]. Cinnamaldehyde (44) was detected at considerable levels in all examined specimens, yet found at its highest levels in commercial pit products (P_2_) reaching up to 7.9% (Figure 1). Cinnamaldehyde exhibits a sweet fruity scent and is the typical aroma compound of cinnamon spice [25], and later was identified in dates (fruit) as a major component [6] which justifies its likely presence herein as well, thus contributes significantly for the roasted date pit aroma.

### 2.2. Multivariate Data Analysis of Headspace SPME-GCMS Volatiles Dataset

**Figure 2:**
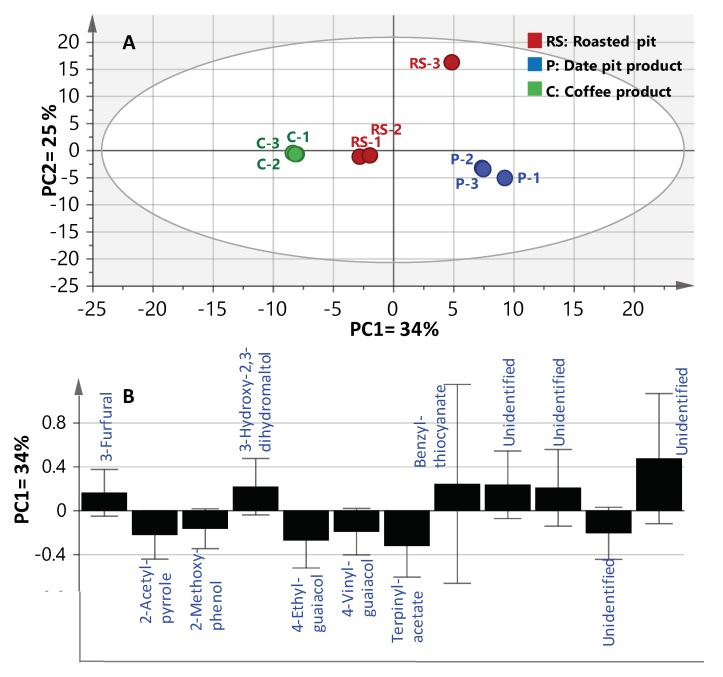
SPME-GCMS based principal component analysis of roasted pit (RS), pit products one and two (merged—P) and coffee product (C) (*n* = 3). The metabolome clusters are located at the distinct positions described by the two vectors of principle component one (PC1) (34%) and PC2 (25%). (**A**) Score Plot of PC1 vs. PC2. (**B**) Loading plot for PC1 and PC2.

To better visualize the subtle volatile differences between roasted coffee and date pits in a rather holistic manner, multivariate data analyses were employed. Principal component analysis (PCA) is an unsupervised clustering process for identifying patterns in data, via reducing the number of dimensions. It can define a limited number of principal components which describe independent variation in the results [26]. In the present study, PCA was first applied on the GCMS volatiles abundance dataset to classify aroma profiles of roasted coffee versus date pits; i.e., R, and P_1_ and P_2_ (merged together as one class denoted P), were analyzed with respect to their chemical composition and to determine markers unique for each specimen. The volatilome clusters were located at different points in the two-dimensional space prescribed by two vectors; that is, principal component one (PC1) accounted for 34% of variation between samples, and PC2 explained 25% of the variance (Figure 2). Inspection of the score plot (Figure 2A) revealed that most of the roasted pit samples were placed on the right side of PC1 (positive score values), whereas coffee specimens were placed on the left side (negative score values). Examination of the loading plot (Figure 2B) revealed key aroma variables referring to MS signals of 2-acetyl-pyrrole (12), terpinyl-acetate (33) and the two aromatics; viz., 2-methoxy-phenol (35), 4-ethyl-guaiacol (37) and 4-vinyl-guaiacol (38) were all found enriched in roasted coffee. In contrast, furans/pyrans; viz., 3-furfural (1), 3-hydroxy-2,3-dihydro-maltol (10) and benzyl-thiocyanate (41) were much more abundant in roasted pit samples (RS and P_2_), suggesting that the latter volatiles could be considered key odorants responsible for the roasted pits’ malty, spicy and caramel like aromas [9,13,22]. 

In spite of the clear separation observed in PCA analysis, metabolite markers were further confirmed by constructing a supervised orthogonal projection to latent structures-discriminant analysis (OPLS-DA). OPLS-DA has greater potential in the identification of markers by providing the most relevant variables for the differentiation between two sample groups. Coffee samples were modelled against all roasted pit samples; viz., RS, and P_1_ and P_2_ (merged together as one class denoted as P) using OPLS-DA. The derived score plot showed a clear separation between both samples (Supplementary Materials, Figure S1), with R^2^ = 0.97 (explaining 97% of the total variance), and a prediction goodness parameter, *Q^2^* = 0.85 (Supplementary Materials, Figure S1A). The corresponding derived S-plot compares the variable magnitude against its reliability, as displayed in Figure S1B, where axes plotted from the predictive component are the covariance p(1) against the correlation p(cor)(1). For the indication of plots with retention time m/z values, a cut-off value of *p* < 0.05 was used. Compared with coffee specimens, roasted pit samples were found to be particularly enriched in benzyl-thiocyanate (41) as a unique marker, typical of a pungent aroma and bitter taste [22], and in agreement with PCA results.

### 2.3. GCMS Analysis of Non-Volatile Primary Metabolites Post Silylation

**Figure 3:**
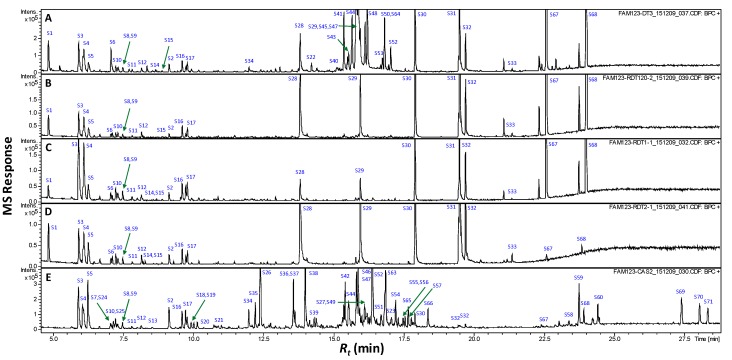
GCMS chromatograms (*R_t_* 5–28 min) of silylated methanol extract from raw pits (**A**), roasted pits (**B**), pit product one (**C**), pit product two (**D**) and coffee product (**E**). The corresponding metabolite names for peaks are shown in Table 2.

Primary metabolites accounting for roasted pit or coffee nutritional value or gustatory attributes (i.e., sugars, and organic, fatty and amino acids) were further profiled using GC/MS post silylation. The analysis’s results led to the detection of 71 metabolites, as listed in Table 2 with their corresponding GC chromatograms displayed (Figure 3). Major primary metabolites identified included saccharides (mono- and di-), alcohols, and organic, fatty and amino acids, and a few sterols/triterpenes. Clearly, monosaccharides and fatty acids amounted for the major primary metabolite classes in raw pits, as opposed to fatty acids’ abundance in roasted pits. Whereas, mono- and disaccharides (sugars) dominated coffee samples.

#### 2.3.1. Alcohols and Organic Acids

Generally, the presence of carboxylic acids (9.5%–24.3% in date pit versus 18.1% in coffee) accounted for its sourness as in most foodstuff [27]; e.g., glycolic acid acetate (S3), lactate (S4), glycolic acid (S5), 3-hydroxypropionic acid (S8), 3-hydroxy-isobutyric acid (S10), acetoacetic acid (S12), benzoic acid (S14), octanoic acid (S15), succinic acid (S16), 2-methyl succinic acid (S17) and methyl maleic acid (S20) were detected in all samples. Oxalic acid (S6) was present in raw pits (RW), whereas benzoic (S14), octanoic (S15) and 3-oxo-glutaric (S22) acids were found exclusively in roasted pit specimens (RS, P_1_ and P_2_); 2-furancarboxylic (S7), 4-hydroxy-butyric (S13), glyceric (S18), fumaric (S19) and 3-deoxytetronic (S21) acids were only present in coffee. 

#### 2.3.2. Amino Acids/Nitrogenous Compounds

Amino acids constituted one major class of coffee’s primary metabolite composition (11%), with sarcosine (S24) and L-pyroglutamic acid (S26) as major components. Those are negligible in all date pits (0.5%–1%). Amino acids react with reducing sugars in the Maillard reaction during the roasting process, yielding an aroma, typical in case of coffee [28]. The low amino acid levels in raw pits (0.5%) could account for the minor amounts of resulting pyrroles, as previously revealed from SPME analysis (Table 1). As expected, caffeine (S27), major alkaloids and bitterness imparting chemicals in coffee [29] were only detected in roasted coffee (Supplementary Materials, Figure S2), being thermostable during the roasting press, unlike other alkaloids. None of those were present in any roasted date pits. The absence of caffeine in roasted date pits while maintaining a similar aroma affirms it as a potential coffee substitute.

#### 2.3.3. Fatty Acids

A major metabolite class present in all unroasted and roasted date pit specimens is fatty acids (19.6%–58.5%), while being nearly absent from coffee. Namely, lauric (S28), myristic (S29), palmitic (S30), oleic (31), stearic (S32) and eicosanoic (S33) acids were found, in agreement with the report of Devshony et al. [2]. The abundance of the monounsaturated omega-9 oleic fatty acid in roasted pits (up to 14.3% of total metabolites and 24.4% of total fatty acid content) makes it a healthy functional food, owing to its high oxidative stability and its potential to lower serum LDL cholesterol [30,31]. 

#### 2.3.4. Sugars

Sugars were found at comparable levels in both raw pits (34.8%) and coffee (34.2%), and are mostly represented by monosaccharides; e.g., 2-deoxy-ribose, ribofuranose, arabinopyranose, ribopyranose, sorbofuranose, fructofuranose, galactopyranose and glucopyranose (S35–S48, S50, S52 and S56), and sugar alcohols (S34 and S54), sugar acids or lactones (S49, S51, S53, S55 and S57). Few disaccharides; viz., lactose (S58) and mannobiose (S59–S60) were also identified in coffee samples (Figure 3 and Table 2). A decrease in sugar levels was detected in roasted pit samples (RS, P_1_ and P_2_) dropping down to 1.5%–1.8%, likely due to sugars’ degradations via Maillard reaction upon roasting. Such a decrease in sugar levels is concurrent with the presence of other bitter chemicals; i.e., benzyl thiocyanate, as revealed from SPME, could account for its less palatable taste than roasted coffee [28]. 

#### 2.3.5. Sterols and Triterpenes 

Few sterols/triterpenes, viz., *β*-sitosterol (S61) and cycloartenol (S62), were detected at relatively moderate levels (7.2%–9.6%) in all date pit specimens and were completely absent from coffee.

### 2.4. Multivariate Data Analyses of Silylated Primary Metabolites

**Figure 4:**
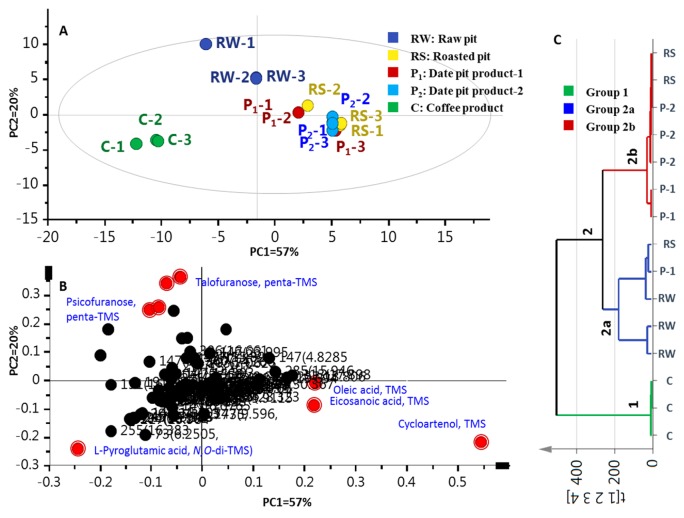
GCMS-post silylation based principal component analysis (PCA) and hierarchical cluster analysis (HCA) of raw pits (RW), roasted pits (RS), pit product one (P_1_), pit product two (P_2_) and coffee product (C) (*n* = 3). (**A**) Score plot of PC1 versus PC2. (**B**) Loading plot for PC1 and PC2. (**C**) HCA of RW, RS, P_1_, P_2_ and C (*n* = 3); the model is colored according to marked groups.

Multivariate data analyses were further employed for specimens’ classification of the primary metabolites dataset’s analogues to the volatiles dataset. The PCA score plot (Figure 4A) revealed 2 confined clusters, with date pit specimens positioned to the right along PC1 (57% of variance) versus another cluster corresponding to coffee specimens exhibiting negative score values along PC1. Examination of the loading plot (Figure 4B) suggested that the MS signal of amino acid L-pyroglutamic acid (S26) accounted for the distant clustering of coffee specimens, whereas monosaccharide sugars S44 and S45 were more enriched in unroasted date pits. The tight clustering of roasted date pits (RS, and P_1_ and P_2_ samples) was attributed to cycloartenol triterpene (S62), along with fatty acids; viz., oleic (S31) and eicosanoic (S33) acids.

Compared to PCA, HCA allows interpretation of the results in a fairly intuitive graphical way. HCA retrieved a dendrogram with three clear clusters of three and 12 samples, referred to as groups 1 and 2, respectively (Figure 4C). Coffee specimens clustered together in group 1, while raw and roasted pit samples (RW, RS, and P_1_ and P_2_) clustered in group 2. Inspection of group 2 showed that (RS) samples are the most closely related to (P_1_) and (P_2_) specimens, being clustered together in a subdivision 2b. However, the clustering of raw pits (RW) with two specimens of roasted pits (RS and P_1_) in branch 2a suggests that HCA cannot clearly distinguish the impact of roasting on date pits. Consequently, a supervised OPLS-DA model was attempted to help classify specimens that failed to separate in HCA, and moreover, to confirm markers revealed from PCA, as in the case of volatile data. 

A model was constructed for roasted coffee against all roasted pit samples (RS, P_1_ and P_2_). The model showed one orthogonal component with *R^2^* = 0.98 and *Q^2^* = 0.97 (Supplementary Materials, Figure S3A). Compared with roasted pit samples (Supplementary Materials, Figure S3B), coffee is particularly enriched in L-pyroglutamic acid (S26). Pyroglutamic acid is a nonessential amino acid that possesses brain-boosting properties through encouraging the memory chemical acetylcholine’s release in the brain [32]. To also assess roasting’s impact on date pits, supervised OPLSDA was applied by modelling unroasted versus roasted date pits (Supplementary Materials, Figure S3C,D). The OPLS-DA score plot (Supplementary Materials, Figure S3C) explained 86% of the total variance (*R^2^* = 0.86), though with less prediction goodness parameter *Q^2^* = 0.76 compared to Figure S3A. The S-plot derived from OPLS-DA (Supplementary Materials, Figure S3D) revealed that compared with unroasted, roasted samples contain more cycloartenol triterpene (S62), although it should be noted that it was found at only trace levels, thus cannot serve as a chemical marker for roasted pits, being a non-validated model component (Table 2).

It can be concluded that analysis of both volatiles and primary metabolites justifies date’s roasted pits’ usage as a popular substitute to coffee beverages, in terms of its similar aroma, high nutritional value though lower sugar levels and no caffeine. However, to be conclusive, evaluation of coffee substitutes needs to be extended beyond metabolite analyses; e.g., to sensory profiles, toxicology and neuroactivity. To provide a first insight into the latter topics, first neuropharmacological bioassays will be discussed in the next sections.

### 2.5. Acute Toxicity Study

The LD_50_ values which assess safety levels of P_1_ and C extracts were estimated to be 1877 ± 39 and 733 ± 39 mg/kg *i.p*. (intra-peritoneal), respectively, indicating P_1_’s larger safety margin [33], and suggesting that P_1_ extract is safer than that of roasted coffee, as revealed in mice.

### 2.6. Neuropharmacological Tests

To further determine whether roasted date product could exhibit any CNS effect similar to that of coffee, extracts of roasted coffee (C) and roasted date pit (P_1_) products were examined for in vivo CNS effects. Considering that chemical analysis (Figure 3 and Figure 4) revealed for the close metabolite composition of products P_1_ and P_2_, neuropharmacological tests were only performed for P_1_.

#### 2.6.1. Phenobarbital Sodium Induced Sleeping Time

Barbiturates are popular hypnotics and sedatives, and can induce sedation in humans and animals through a CNS-depressant effect [34]. In the control groups (1 and 2), phenobarbital sodium (10 mg/kg) produced an intermediate onset and duration of sleep, as indicated by the loss of and regaining of righting reflex subsequently [35]. Pretreatment with commercial date pits (P_1_) in groups 3 and 4 showed no significant reduction in the sleep induction time nor duration. In contrast, pretreatment with (C) coffee extract in groups 5 and 6 markedly and significantly reduced sleeping time and prolonged the duration of sleep in phenobarbital sodium induced mice (Supplementary Materials, Figure S4A and Table S1).

#### 2.6.2. Open Field Test

Another assessed CNS induction test was performed (Figure 2B and Table S2) to confirm results presented in Figure 2A. The locomotive activity was evaluated in an open field test to assess the CNS stimulant property of P_1_ on mice’s motor activity. Locomotive activity reflects alertness and wakefulness of mental activity and a decrease may lead to calming and sedation [36]. Compared to the normal control group, it was found that P_1_ extract exhibited no significant change in mice’s locomotive activity (the number of squares crossed and number of rearings), so showed less significant motor stimulant activity than (C) coffee extract (Supplementary Materials, Figure S4B and Table S2). 

Both bioassays affirmed that commercially-roasted pit product (P_1_) exhibits no coffee-like CNS stimulant property in mice which may be attributed to caffeine’s absence, supporting its use as coffee substitute beverage.

## 3. Materials and Methods 

### 3.1. Plant Material, Animals and Chemicals

Date palm pit variety Majdool (KSA) was obtained from a commercial store in Cairo (Egypt) and subjected to roasting (RS) as described in Section 2, whereas roasted date commercial pit products Nawat-Altamr Coffee “Almasmak Trade^®^” (P_1_) and (P_2_) were purchased from Riyadh (Saudi Arabia). A commercial coffee powder “Hintz^®^, Germany” (C) was used for comparison. The SPME holder and fiber coated with 50 μm/30 μm DVB–CAR–PDMS were supplied by Supelco (Oakville, ON, Canada). Normal saline was purchased from El Nasr Pharmaceutical Chemicals Company (Cairo, Egypt). Phenobarbital sodium and all other chemicals and volatile standards were provided from Sigma Aldrich (St. Louis, MO, USA).

For the biological study, adult male Swiss mice (22 ± 4 g) were purchased from the animal house colony of National Research Centre (NRC), Giza, Egypt. The animals were housed in stainless steel wire–meshed plastic cages under standard conditions of humidity, temperature (25 ± 2 °C), and light/dark cycles (12/12 h). Rodent chow diet and water were allowed ad libitum. All experiments were carried out in accordance with the research protocols established by Research Ethics Committee in the Faculty of Pharmacy, Cairo University and by Medical Research Ethics Committee (MREC) in NRC, which follow the recommendations of the National Institutes of Health Guide for the Care and Use of Laboratory Animals (Ethical Approval Certificate number MP (1589)).

### 3.2. The Preparations of Roasted Date Pits, Commercial Pit Product and Coffee Product Extracts for Chemical and Biological Analyses

For the preparation of home-roasted pit (RS) for subsequent chemical analysis, date palm pits (100 g) were oven roasted at 120 °C for 3 h, with stirring using a magnetic stirrer; roasted pits were then ground into fine powder.

For biological studies, commercial products, viz., roasted date pits (P_1_) and coffee (C), 150 g of each were extracted with 100% methanol by cold maceration until complete exhaustion. The methanol extract was evaporated under reduced pressure at a temperature not exceeding 40 °C until dryness, and the solvent was further evaporated under reduced pressure to yield the corresponding extracts (4.6 and 106.5 g) respectively.

### 3.3. SPME Volatiles Analysis 

Headspace volatiles analysis using SPME was adopted from Farag and Wessjohann, (2012) [37], with few modifications. Three grams of each sample (RS, and P_2_ and C) were placed inside 20 mL clear glass vials. The compound (*Z*)-3-hexenyl acetate, absent from pit sample volatiles, was used as an internal standard (IS). Vials were then immediately capped and placed on a temperature-controlled tray for 30 min at 50 °C with the SPME fiber inserted into the headspace above the sample. A system blank containing no plant material was run as a control.

### 3.4. GCMS Headspace Volatile Analysis

SPME fiber was desorbed at 210 °C for 1 min in the injection port of a Shimadzu Model GC-17A gas chromatograph interfaced with a Shimadzu model QP-5000 mass spectrometer (Kyoto, Japan). Volatiles were separated on a DB5-MS column (J&W Scientific, Santa Clara, CA, USA). Injections were made in the splitless mode for 30 s. The gas chromatograph was operated under the conditions described in Farag and Wessjohann, (2012) [37]. The HP quadrupole mass spectrometer was operated in the electron ionization mode at 70 eV. The scan range was set at 40–500 *m*/*z*. Peaks were first deconvoluted using AMDIS software (www.amdis.net) and identified by its retention indices (RI) relative to n-alkanes (C6–C20), mass spectra matching to NIST, the WILEY library database and with authentic standards when available.

### 3.5. GCMS Analysis of Silylated Primary Metabolites 

For analysis of primary metabolites in methanol extracts of the different samples (RW, RS, P_1_, P_2_ and C), a derivatization step was performed prior to analysis, as described in Farag et al., (2015) [38]. Briefly, 50 μL of dried methanol extract was mixed with 100 μL of *N*-methyl-*N*-(trimethylsilyl)-trifluoroacetamide (MSTFA) and incubated at 60 °C for 45 min. Samples were equilibrated at 28 °C and subsequently analyzed using GCMS a Shimadzu model QP-5000 mass spectrometer (Kyoto, Japan). Silylated derivatives were separated on an Rtx-5MS column. Injections were made in a (1:15) split mode and the GC was operated under the conditions mentioned in Farag et al., (2015) [38]. The HP quadrupole mass spectrometer was operated in the electron ionization mode at 70 eV. The scan range was set at 50–650 *m*/*z*. Silylated compounds were identified as previously described under GCMS volatile analysis, and their contents were determined based on peak areas relative to summed peak areas of total identified metabolites within each specimen. 

### 3.6. Multivariate Data Analyses

Principal component analysis (PCA), hierarchical clustering analysis (HCA) and partial least squares-discriminant analysis (OPLS-DA) were performed on the GCMS datasets, using the program SIMCA-P Version 13.0 (Umetrics, Umeå, Sweden). All variables were mean centered and scaled to Pareto variance. The distance to the model (DModX) test was used to verify the presence of outliers and to evaluate whether a submitted sample fell within the model applicability domain.

### 3.7. Acute Toxicity Study

The toxicity study was carried out to determine the LD_50_ values of each product extract, date pits (P_1_) and coffee (C), using the graphical method of Litchfield and Wilcoxon, (1949) in mice [39]. Seven groups of six mice (25 g) each received an extract in 7 doses, starting from no death to 100% mortality; 100, 500, 1000, 3000, 5000, 7500 and 10,000 mg/kg, *i.p*. The control group received normal saline (5 mL/kg, *i.p*.). Signs of toxicity and mortality within 24–72 h were recorded and the LD_50_ was calculated from the log-probit graph.

### 3.8. Neuropharmacological Tests

#### 3.8.1. Treatment Schedule

Acute (150 mg/kg single dose) and subacute (150 mg/kg/day for 7 days) studies were performed to evaluate the neuropharmacological effects of P_1_ against C extract as a reference. The neuropharmacological activity was evaluated using the open field test and phenobarbital sodium induced sleeping time test. The animals were divided into six groups (*n* = 6) as follows: groups 1 and 2; served as negative controls, receiving normal saline (5 mL/kg, *p.o*.), for one day (acute study) and 7 d (subacute study), respectively; groups 3 and 4 were administered P_1_ extract (150 mg/kg, *p.o*.) for acute and subacute studies respectively; and finally, groups 5 and 6 served as reference groups, receiving (C) extract (150 mg/kg, *p.o*.) in the same way as groups 3 and 4 did, at an equal dose of 150 mg/kg, *p.o*., for 1 and 7 d respectively.

#### 3.8.2. Phenobarbital Sodium Induced Sleeping Time

A phenobarbital sodium induced sleeping time test was carried out following the protocol of Williamson et al., (1996) [40]. Thirty minutes after treatments, phenobarbital sodium was given *i.p*. at a dose of 10 mg/kg to all animal groups, and then each animal was kept in an individual cage under observation. The latency to the loss of righting reflex (i.e., onset of action or induction time) and the time required to recover righting reflex or awakening (i.e., duration of action or sleeping time) in minutes for each group were recorded [41].

#### 3.8.3. Open Field Test

Locomotor activity was evaluated by applying the Open Field Test. The open field apparatus consisted of a wooden box (60 × 60 × 60 cm); the arena of the open field was divided into 16 squares (15 × 15 cm): the four inner squares in the center and 12 squares on the periphery along the walls. The experimental room was a sound attenuated, shaded room. After 60 min of oral treatments, animals were placed individually in one of the corner squares and the following behavioral parameters were scored for a period of 5 minutes: (1) the number of squares crossed (as a measure of distance travelled), and (2) the number of rearings (number of times the animal stood on hind legs) [42].

#### 3.8.4. Statistical Analysis

All results were expressed as the mean ± standard error of mean (SEM). The results were analyzed for statistical significance by two-way analyses of variance (ANOVA) followed by Bonferroni post hoc tests (*p* < 0.05).

## 4. Conclusions

The metabolite profiles, nutritional, safety and neuropharmacological aspects of coffee substitutes from the roasted Majdool variety date pit were assessed in this study. SPME headspace analysis revealed the abundance of furans, pyrans, terpenoids and sulfur compounds in roasted date pit, whereas pyrroles and caffeine, typical metabolites of roasted coffee, were absent. Key odorants of roasted date pits were identified for the first time in this study. There is also evidence that the commercial date pit product was spiked with additives; namely, mono- or sesquiterpenoids. Moreover, primary metabolites accounting for the sensory and nutritive values were evaluated in roasted pits using GC/MS, among which fatty acids were the most abundant class in pits, compared to sugars in coffee. Biological tests affirmed that commercial date pit product (P_1_) shows no CNS stimulant property. The absence of caffeine in roasted date pit concurrent with its enrichment in nutrients, viz., monounsaturated fatty acids, makes it a healthy, functional food beverage for consumers with caffeine concerns, as additionally revealed by its higher safety margin; i.e., LD_50_ dose. It should be noted that these are results which are specific to two roasted date commercial preparations of Saudi origin. More results need to be presented for profiling from other products or from other date varieties to be conclusive.

## Figures and Tables

**Table 1:** Relative percentage of volatile components in roasted pits (RS), pit product two (P_2_) and coffee product (C) using headspace SPME-GCMS. Each value represents mean ± S.D. (*n* = 3).

Peak No.	*R_t_* (min)	RI	Compound	RS	P _2_	C
Furans						
1	5.74	840.5	3-Furfural	7.58 ± 5.84	0.05 ± 0.02	0.02 ± 0.02
2	7.18	912.0	2-Acetylfuran	0.45 ± 0.02	0.13 ± 0.09	0.02 ± 0.02
3	7.31	919.2	Dihydro-4-methyl-2(3H)-furanone	0.14 ± 2.46	0.12 ± 0.03	0.14 ± 0.10
4	8.11	965.2	5-Methyl-2-furaldehyde	15.92 ± 1.20	4.88 ± 5.82	3.73 ± 0.96
5	8.57	991.0	Furfuryl acetate	1.19 ± 0.10	0.27 ± 0.36	4.21 ± 0.84
6	9.96	1079.5	2-Furfurylfuran	1.00 ± 0.05	0.06 ± 0.08	2.02 ± 0.18
7	11.34	1174.2	5-Methyl-2-furfurylfuran	-	0.33 ± 0.44	1.16 ± 0.35
8	13.00	1298.2	Difurfuryl ether	0.21 ± 0.15	0.02 ± 0.00	1.33 ± 0.19
Total furans				26.50	5.87	12.71
Pyrans						
9	10.39	1128.1	Maltol	9.86 ± 0.04	8.60 ± 10.92	0.86 ± 0.02
10	11.14	1160.2	3-Hydroxy-2,3-dihydromaltol	2.78 ± 1.15	-	-
11	15.07	1467.2	Benzo-*α*-pyrone	-	0.05 ± 0.00	-
Total pyrans				12.64	8.65	0.86
Pyrroles						
12	10.05	1085.2	2-Acetyl-pyrrole	1.00 ± 0.44	-	4.10 ± 1.46
13	11.43	1180.3	1-(2-Furanyl-methyl)-1H-pyrrole	-	-	1.60 ± 0.18
Total pyrroles				1.00	-	5.70
Lactones						
14	9.34	1039.7	Cyclotene (Maple lactone)	1.47 ± 0.12	-	1.58 ± 0.27
Total lactones				1.47	-	1.58
Monoterpenoid hydrocarbons						
15 ^a^	9.17	1029.1	*α*-Phellandrene	-	2.22 ± 1.80	1.13 ± 3.03
Total monoterpenoid hydrocarbons				-	2.22	1.13
Sesquiterpenoid hydrocarbons						
16	14.01	1380.5	*α*-Copaene	-	0.30 ± 0.37	-
17	14.58	1427.4	(*E*,*β*)-Farnesene	-	1.11 ± 0.96	-
18 ^a^	15.15	1473.9	*α*-Humulene	-	0.57 ± 0.57	-
19	15.20	1478.0	Curcumene	-	3.65 ± 3.01	-
20 ^a^	15.36	1491.1	*α*-Zingiberene	-	2.61 ± 2.50	-
21 ^a^	15.53	1504.5	*β*-Bisabolene	-	2.63 ± 2.99	-
22	15.58	1508.0	*δ*-Selinene	-	0.40 ± 0.07	-
23	15.77	1520.7	*β*-Sesquiphellandrene	-	1.76 ± 1.68	-
24	15.95	1533.6	Calamenene	-	-	-
Total sesquiterpenoid hydrocarbons				-	13.04	-
Oxygenated monoterpenoids						
25 ^a^	9.25	1034.0	Cineole	-	1.09 ± 1.13	8.22 ± 0.81
26 ^a^	10.25	1097.8	Linalool	-	2.45 ± 2.84	1.94 ± 0.39
27 ^a^	11.14	1160.3	*p*-Menthone	0.42 ± 0.01	0.04 ± 0.02	-
28	11.44	1181.4	Isopulegone	-	0.03 ± 0.05	1.60 ± 0.15
29	11.72	1200.6	*α*-Terpineol	-	0.85 ± 0.85	1.10 ± 0.08
30 ^a^	12.29	1243.9	Linalyl acetate	-	-	6.58 ± 0.01
31	12.34	1247.9	Pulegone	0.68 ± 0.19	11.67 ± 11.18	-
32 ^a^	12.39	1251.8	Carvone	-	1.33 ± 1.01	-
33	13.62	1348.2	Terpinyl acetate	0.04 ± 0.05	0.29 ± 0.25	26.21 ± 2.86
34 ^a^	13.73	1357.8	Eugenol	0.11 ± 0.06	0.36 ± 0.21	-
Total oxygenated monoterpenoids				1.25	18.09	45.65
Phenolics						
35	10.13	1089.9	2-Methoxy phenol	0.22 ± 0.04	0.24 ± 0.18	8.44 ± 2.07
36	10.92	1144.9	Benzyonitrile	3.45 ± 1.22	-	-
37	12.75	1279.2	4-Ethylguaiacol	0.11 ± 0.07	0.00 ± 0.01	9.96 ± 1.62
38	13.25	1318.4	4-Vinylguaiacol	0.10 ± 0.07	-	3.92 ± 1.61
Total phenolics				3.87	0.25	22.32
Sulfur compounds						
39	11.63	1194.2	2-Propionylthiophene	-	-	0.29 ± 0.15
40	11.93	1216.7	2-Furfuryl methyl disulfide	-	-	0.09 ± 0.02
41	13.92	1372.7	Benzyl-thiocyanate	6.42 ± 3.79	42.34 ± 59.82	-
Total sulfur compounds				6.42	42.34	0.37
Aldehydes/ketones						
42 ^a^	10.31	1101.7	Nonanal	2.20 ± 1.01	0.01 ± 0.01	0.37 ± 0.15
43 ^a^	11.73	1201.9	*n*-Decanal	1.13 ± 0.76	-	-
44 ^a^	12.83	1284.4	(*E*)-Cinnamaldehyde	1.90 ± 0.40	7.86 ± 10.28	5.68 ± 2.07
45	12.87	1287.9	2-Undecanone	1.03 ± 0.47	1.03 ± 0.75	1.70 ± 0.46
Total aldehydes/ketones				6.25	8.89	7.75
Hydrocarbons						
46	11.56	1189.5	2-Methylundecane	0.87 ± 0.04	0.15 ± 0.05	-
47	14.20	1396.3	4-Methyltridecane	1.10 ± 0.04	0.03 ± 0.05	-
Total hydrocarbons				1.97	0.18	-
Unidentified volatiles						
48	12.17	1235.4	Unknown	-	0.62 ± 0.61	-
49	20.06	1766.7	Unknown	5.96 ± 3.80	-	-
50	20.62	1795.7	Unknown	17.04 ± 7.90	-	-
51	20.85	1807.9	Unknown	0.33 ± 0.10	-	3.04 ± 1.28
52	21.25	1828.6	Unknown	15.30 ± 11.24	-	-
Total unidentified volatiles				38.63	0.62	3.04

RI, Kovat index; ^a^ represents volatiles confirmed using authentic standards.

**Table 2:** Relative percentages of silylated primary metabolites in raw pits (RW), roasted pits (RS), pit product one (P_1_), pit product two (P_2_) and coffee product (C) using GCMS. Each value represents mean ± S.D. (*n* = 3).

Peak No.	*R_t_* (min)	RI	Compound	RW	RS	P_1_	P_2_	C
Alcohols								
S1	4.3	971.6	Ethylene glycol, di-TMS	3.22 ± 0.63	5.19 ± 1.83	5.97 ± 2.36	4.07 ± 2.25	0.24 ± 1.02
S2	9.12	1281.9	Glycerol, tri-TMS	0.21 ± 0.04	0.12 ± 0.12	0.19 ± 0.04	0.32 ± 0.07	0.50 ± 0.15
Total alcohols				3.43	5.30	6.16	4.39	0.74
Acids and lactones								
S3	5.91	1051.2	Glycolic acid acetate, TMS	2.38 ± 0.95	3.03 ± 0.01	7.96 ± 1.18	3.14 ± 3.96	2.65 ± 1.43
S4	6.08	1062.0	Lactic acid, di-TMS	1.41 ± 0.64	1.45 ± 0.55	3.50 ± 0.50	2.08 ± 1.72	2.54 ± 0.83
S5	6.25	1076.1	Glycolic acid, di-TMS	1.27 ± 0.51	1.95 ± 0.54	2.81 ± 0.82	4.26 ± 1.24	5.15 ± 1.88
S6	7.06	1134.6	Oxalic acid, di-TMS	0.34 ± 0.32	0.04 ± 0.01	0.04 ± 0.17	0.03 ± 0.08	0.01 ± 0.07
S7	7.07	1135.1	2-Furancarboxylic acid, TMS	0.03 ± 0.02	0.04 ± 0.01	0.11 ± 0.02	0.22 ± 0.05	0.44 ± 0.11
S8	7.23	1146.4	3-Hydroxypropionic acid, di-TMS	0.60 ± 0.27	0.94 ± 0.24	1.34 ± 0.39	0.93 ± 0.59	0.46 ± 0.27
S9	7.30	1151.9	Pantolactone, TMS	0.25 ± 0.13	0.35 ± 0.09	0.52 ± 0.14	0.37 ± 0.24	0.19 ± 0.12
S10	7.47	1163.5	3-Hydroxyisobutyric acid, di-TMS	0.74 ± 0.35	0.96 ± 0.28	1.81 ± 0.38	1.08 ± 0.86	0.68 ± 0.36
S11	7.81	1187.9	Cyclohexane-carboxylic acid, TMS	0.13 ± 0.06	0.19 ± 0.05	0.31 ± 0.07	0.25 ± 0.14	0.12 ± 0.09
S12	8.14	1211.3	Acetoacetic acid, di-TMS	0.14 ± 0.05	0.37 ± 0.07	0.37 ± 0.18	0.49 ± 0.16	0.13 ± 0.19
S13	8.53	1239.3	4-Hydroxybutyric acid, di-TMS	0.05 ± 0.04	0.03 ± 0.01	0.07 ± 0.02	0.06 ± 0.04	0.10 ± 0.02
S14	8.69	1250.9	Benzoic acid, TMS	0.19 ± 0.11	0.23 ± 0.06	0.32 ± 0.09	0.33 ± 0.14	0.05 ± 0.13
S15	8.88	1264.5	Octanoic acid, TMS	0.11 ± 0.04	0.17 ± 0.05	0.21 ± 0.07	0.18 ± 0.09	0.04 ± 0.06
S16	9.60	1316.9	Succinic acid, di-TMS	0.99 ± 0.38	1.78 ± 0.43	2.72 ± 0.79	3.39 ± 1.23	1.88 ± 1.39
S17	9.77	1330.2	2-Methyl succinic acid, di- TMS	0.56 ± 0.18	1.10 ± 0.27	1.78 ± 0.51	1.47 ± 0.81	1.86 ± 0.49
S18	9.92	1340.9	Glyceric acid, tri-TMS	0.05 ± 0.00	0.03 ± 0.03	0.02 ± 0.02	0.05 ± 0.01	0.14 ± 0.02
S19	10.02	1348.5	Fumaric acid, di-TMS	0.01 ± 0.00	0.01 ± 0.01	0.01 ± 0.00	0.01 ± 0.00	0.20 ± 0.01
S20	10.14	1357.5	Methyl maleic acid, di-TMS	0.09 ± 0.03	0.15 ± 0.04	0.28 ± 0.06	0.23 ± 0.13	1.04 ± 0.09
S21	11.00	1423.1	3-Deoxytetronic acid, di TMS	0.01 ± 0.00	0.14 ± 0.01	0.08 ± 0.08	0.05 ± 0.04	0.42 ± 0.02
S22	14.21	1688.7	3-Oxo-glutaric acid, tri-TMS	0.20 ± 0.16	0.01 ± 0.03	0.00 ± 0.09	0.00 ± 0.04	0.00 ± 0.04
Total acids and lactones				9.56	12.94	24.25	18.64	18.10
Phenolic acid								
S23	17.07	1961.3	Hydrocaffeic acid, tri-TMS	-	-	0.01 ± 0.00	0.01 ± 0.00	0.12 ± 0.00
Total phenolic acid				-	-	0.01	0.01	0.12
Amino acids and other nitrogenous compounds								
S24	7.10	1137.2	Sarcosine, *N*,*O*-di-TMS	0.20 ± 0.06	0.49 ± 0.09	0.57 ± 0.24	0.55 ± 0.24	0.19 ± 0.18
S25	7.15	1140.5	3-Pyridinol, TMS	0.01 ± 0.01	0.01 ± 0.00	0.05 ± 0.00	0.06 ± 0.03	0.93 ± 0.03
S26	12.38	1533.6	L-Pyroglutamic acid, *N*,*O*-di-TMS	0.26 ± 0.04	0.11 ± 0.16	0.16 ± 0.06	0.41 ± 0.06	10.82 ± 0.21
S27	16.10	1865.2	Caffeine	0.03 ± 0.02	-	0.00 ± 0.01	0.00 ± 0.01	3.61 ± 0.00
Total amino acids				0.46	0.60	0.73	0.96	11.01
Fatty acids								
S28	13.81	1653.8	Lauric acid, TMS	2.37 ± 0.65	7.58 ± 1.21	4.53 ± 3.85	7.62 ± 1.75	0.02 ± 2.97
S29	15.95	1850.2	Myristic acid, TMS	2.23 ± 0.59	5.39 ± 1.16	3.50 ± 2.62	5.89 ± 1.18	0.04 ± 2.41
S30	17.90	2045.1	Palmitic acid, TMS	3.45 ± 0.89	7.70 ± 1.81	6.14 ± 3.70	9.49 ± 2.17	0.60 ± 3.86
S31	19.49	2205.5	Oleic acid, TMS	3.74 ± 1.17	11.51 ± 1.82	7.09 ± 5.79	14.28 ± 2.75	0.08 ± 5.98
S32	19.69	2226.5	Stearic acid, TMS	1.28 ± 0.43	3.32 ± 0.60	2.14 ± 1.62	4.20 ± 0.79	0.19 ± 1.78
S33	21.36	2394.5	Eicosanoic acid, TMS	6.59 ± 2.05	15.26 ± 3.21	16.62 ± 7.32	17.06 ± 6.87	4.31 ± 5.76
Total fatty acids				19.64	50.77	40.01	58.54	5.24
Sugars								
S34	11.97	1499.6	L-Threitol, tetra-TMS	0.38 ± 0.15	0.14 ± 0.16	0.29 ± 0.01	0.25 ± 0.14	1.26 ± 0.12
S35	12.21	1518.9	2-Deoxy-D-ribose, tri-TMS	0.01 ± 0.00	0.03 ± 0.01	0.02 ± 0.01	0.05 ± 0.01	2.50 ± 0.02
S36	13.56	1632.7	Ribofuranose, tetra-TMS	0.01 ± 0.00	0.01 ± 0.01	0.02 ± 0.01	0.03 ± 0.01	3.24 ± 0.01
S37	13.62	1637.3	Arabinopyranose, tetra-TMS	-	0.01 ± 0.00	0.01 ± 0.00	0.02 ± 0.00	0.59 ± 0.01
S38	13.98	1669.2	Ribopyranose, tetra-TMS	0.01 ± 0.01	0.01 ± 0.00	0.01 ± 0.00	0.01 ± 0.00	3.61 ± 0.01
S39	14.30	1696.9	Ribofuranose, tetra-TMS isomer	0.36 ± 0.21	0.11 ± 0.10	0.09 ± 0.06	0.10 ± 0.02	0.43 ± 0.04
S40	15.21	1779.9	Sorbofuranose, penta-TMS	0.53 ± 0.43	0.01 ± 0.07	0.01 ± 0.23	0.01 ± 0.11	0.57 ± 0.11
S41	15.36	1794.1	Fructofuranose, penta-TMS	1.55 ± 0.75	0.28 ± 0.57	0.10 ± 0.24	0.21 ± 0.24	1.54 ± 0.02
S42	15.41	1798.6	Psicopyranose, penta-TMS	0.03 ± 0.02	0.02 ± 0.01	0.01 ± 0.00	0.01 ± 0.00	1.11 ± 0.01
S43	15.49	1806.1	Tagatofuranose, penta-TMS	0.46 ± 0.28	0.02 ± 0.12	0.02 ± 0.13	0.02 ± 0.06	0.13 ± 0.06
S44	15.65	1821.7	Talofuranose, penta-TMS	6.73 ± 2.96	0.18 ± 2.66	0.04 ± 1.53	0.09 ± 1.31	0.28 ± 0.78
S45	15.79	1835.4	Psicofuranose, penta-TMS	5.68 ± 2.39	0.21 ± 2.33	0.30 ± 1.24	0.27 ± 1.02	1.77 ± 0.51
S46	15.81	1837.3	Talopyranose, penta-TMS	1.30 ± 0.30	0.01 ± 0.71	0.01 ± 0.35	0.02 ± 0.35	2.40 ± 0.19
S47	15.87	1843.2	Talofuranose, penta-TMS isomer	5.01 ± 3.29	0.03 ± 1.21	0.01 ± 1.65	0.03 ± 0.85	1.02 ± 0.81
S48	16.12	1867.1	Galactopyranose, penta-TMS	3.53 ± 1.25	0.05 ± 1.61	0.01 ± 0.82	0.02 ± 0.80	0.28 ± 0.46
S49	16.30	1884.0	3-Deoxy-arabino-hexonic acid, penta-TMS	4.90 ± 1.60	0.26 ± 2.33	0.26 ± 1.05	0.24 ± 1.04	5.49 ± 0.47
S50	16.66	1920.1	Psicose, penta-TMS, TMS-oxy oxime	0.57 ± 0.56	0.00 ± 0.01	0.00 ± 0.32	0.00 ± 0.18	0.04 ± 0.16
S51	16.69	1922.0	Glucuronic acid γ-lactone TMS, trimelthyoxyoxime	0.06 ± 0.03	0.01 ± 0.02	0.01 ± 0.01	0.01 ± 0.01	0.27 ± 0.00
S52	16.81	1934.9	Glucose, penta-TMS	1.23 ± 0.60	0.01 ± 0.45	0.00 ± 0.31	0.00 ± 0.23	0.02 ± 0.16
S53	17.03	1957.5	Gulonic acid, 1,4-lactone, tetra-TMS	1.32 ± 0.82	0.10 ± 0.35	0.01 ± 0.37	0.05 ± 0.20	0.21 ± 0.16
S54	17.23	1977.1	Mannitol, hexa-TMS	0.32 ± 0.25	0.08 ± 0.05	0.13 ± 0.10	0.13 ± 0.04	1.59 ± 0.05
S55	17.47	2002.1	Mannonic acid, 1,4-lactone, tetra-TMS	0.14 ± 0.08	0.15 ± 0.05	0.07 ± 0.05	0.17 ± 0.01	0.27 ± 0.08
S56	17.54	2008.7	Glucopyranose, penta-TMS	0.55 ± 0.37	0.03 ± 0.13	0.02 ± 0.18	0.03 ± 0.08	0.72 ± 0.08
S57	17.77	2031.7	Glucuronic acid, penta-TMS	0.02 ± 0.01	0.01 ± 0.01	0.01 ± 0.00	0.02 ± 0.00	0.70 ± 0.01
S58	23.74	2635.0	Lactose, octa-TMS (isomer 2)	0.08 ± 0.06	0.01 ± 0.02	0.01 ± 0.02	0.01 ± 0.01	1.21 ± 0.01
S59	23.91	2652.8	3-Mannobiose, octa-TMS	0.02 ± 0.01	0.02 ± 0.00	0.02 ± 0.01	0.01 ± 0.01	0.32 ± 0.00
S60	24.41	2703.1	2-Mannobiose, octa-TMS	0.02 ± 0.01	0.01 ± 0.01	0.07 ± 0.01	0.01 ± 0.04	2.68 ± 0.02
Total sugar				34.81	1.79	1.54	1.81	34.23
Sterols and triterpenes								
S61	30.37	3304.6	*β*-Sitosterol, TMS	0.71 ± 0.13	2.03 ± 0.41	2.65 ± 1.02	2.98 ± 1.16	0.05 ± 1.09
S62	31.50	3419.0	Cycloartenol, TMS	2.37 ± 0.65	7.58 ± 1.21	4.53 ± 1.85	4.87 ± 1.75	0.00 ± 1.59
Total sterols/triterpenes				3.08	9.61	7.18	7.85	0.05
Unidentified								
S63	16.38	1892.4	Unknown	0.01 ± 0.00	0.01 ± 0.01	0.04 ± 0.00	0.00 ± 0.02	5.98 ± 0.01
S64	16.73	1927.3	Unknown	2.14 ± 0.87	0.03 ± 0.90	0.01 ± 0.49	0.02 ± 0.44	4.34 ± 0.26
S65	17.65	2020.4	Unknown	-	-	-	0.01 ± 0.00	0.36 ± 0.01
S66	18.36	2091.4	Unknown	0.25 ± 0.37	0.00 ± 0.08	0.00 ± 0.19	0.00 ± 0.09	1.09 ± 0.09
S67	22.58	2518.2	Unknown	7.22 ± 12.18	10.40 ± 3.50	11.28 ± 4.58	0.86 ± 4.21	0.08 ± 2.05
S68	24.00	2661.0	Unknown	2.82 ± 4.22	4.08 ± 0.99	5.07 ± 1.83	0.46 ± 2.15	0.79 ± 0.90
S69	27.39	3003.6	Unknown	0.01 ± 0.00	0.01 ± 0.01	0.02 ± 0.00	0.02 ± 0.01	3.28 ± 0.01
S70	28.03	3068.6	Unknown	-	0.01 ± 0.00	0.01 ± 0.00	0.01 ± 0.00	0.99 ± 0.00
S71	28.32	3098.1	Unknown	0.66 ± 0.59	3.52 ± 0.05	0.63 ± 1.86	2.31 ± 0.93	1.06 ± 0.71
Total unidentified				13.11	18.06	17.07	3.68	17.97

TMS: trimethyl-silyl; RI, Kovat index.

## Supplementary Materials

The following are available online. Figure S1: A. OPLS-DA score plot and B. loading S-plots derived from coffee (C) samples modelled against roasted pit samples i.e., roasted pit (RS) and products (P) analysed by SPME-GCMS; Figure S2: Representative GCMS-post silylation overlaid chromatograms (*R_t_* 15.9–16.2 min) showing the absence of caffeine in roasted pit (RS) and pit product (P) samples; Figure S3: OPLS-DA score plot and B. loading S-plots derived from coffee samples modelled against roasted pit samples (RS, P_1_, and P_2_) analysed by GCMS-post silylation (*n* = 3). C. OPLS-DA score plot and D. loading S-plots derived from raw pit samples modelled against roasted pit samples (RS, P_1_, and P_2_) analysed by GCMS-post silylation; Figure S4: A. Effect of date pit product (P_1_) and coffee product (C) extracts on the onset and duration of sleep time during phenobarbital sodium induced sleeping test. B. Effect of date pit product (P_1_) and coffee product (C) extracts on the onset and duration of sleep time during phenobarbital sodium induced sleeping test; Table S1: Effect of date pit product (P) and coffee product (C) extracts on the onset and duration of sleep time during phenobarbital sodium induced sleeping test; Table S2: Effect of date pit product (P) and coffee product (C) on the number of squares crossed by mice and number of rearing during Open Field Test.
