# Supplementary material for: Sensory Metabolite Profiling in a Date Pit Based Coffee Substitute and in Response to Roasting as Analyzed via Mass Spectrometry Based Metabolomics
Source: Molecules. 2019 Sep 17;24(18):3377. doi: 10.3390/molecules24183377 (PMC6767185; doi:10.3390/molecules24183377)
Supplement: Supplementary file 1 [file molecules-24-03377-s001.pdf]

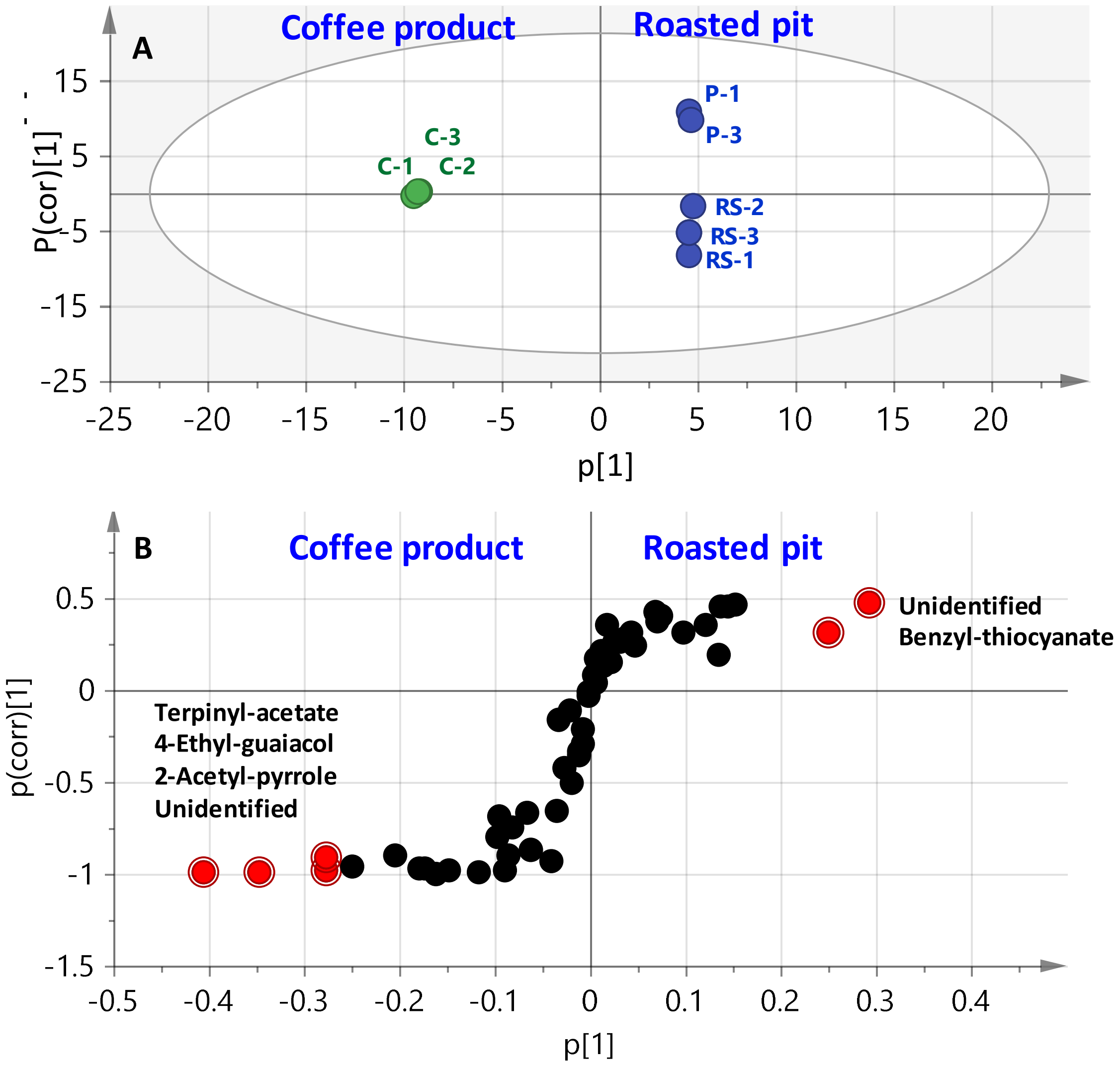

A
Coffee product
Roasted pit
C-3
C-1
C-2
P-1
P-3
RS-2
RS-3
RS-1
P(cor)[1]
p[1]
B
Coffee product
Roasted pit
Unidentified
Benzyl-thiocyanate
Terpinyl-acetate
4-Ethyl-guaiacol
2-Acetyl-pyrrole
Unidentified
p(corr)[1]
p[1]

Figure 1. A. OPLS-DA score plot and **B.** loading S-plots derived from coffee (C) samples ( ) modelled against roasted pit samples *i.e.*, roasted pit (RS) and products (P) ( ) analysed by SPME-GCMS (n =3). The S-plot showing the covariance p[1] against the correlation p(cor)[1] of the variables of the discriminating component of the OPLS-DA model. Cut-off values of $P<0.05$ were used; variables selected are highlighted in the S-plot and identifications are discussed in the text.

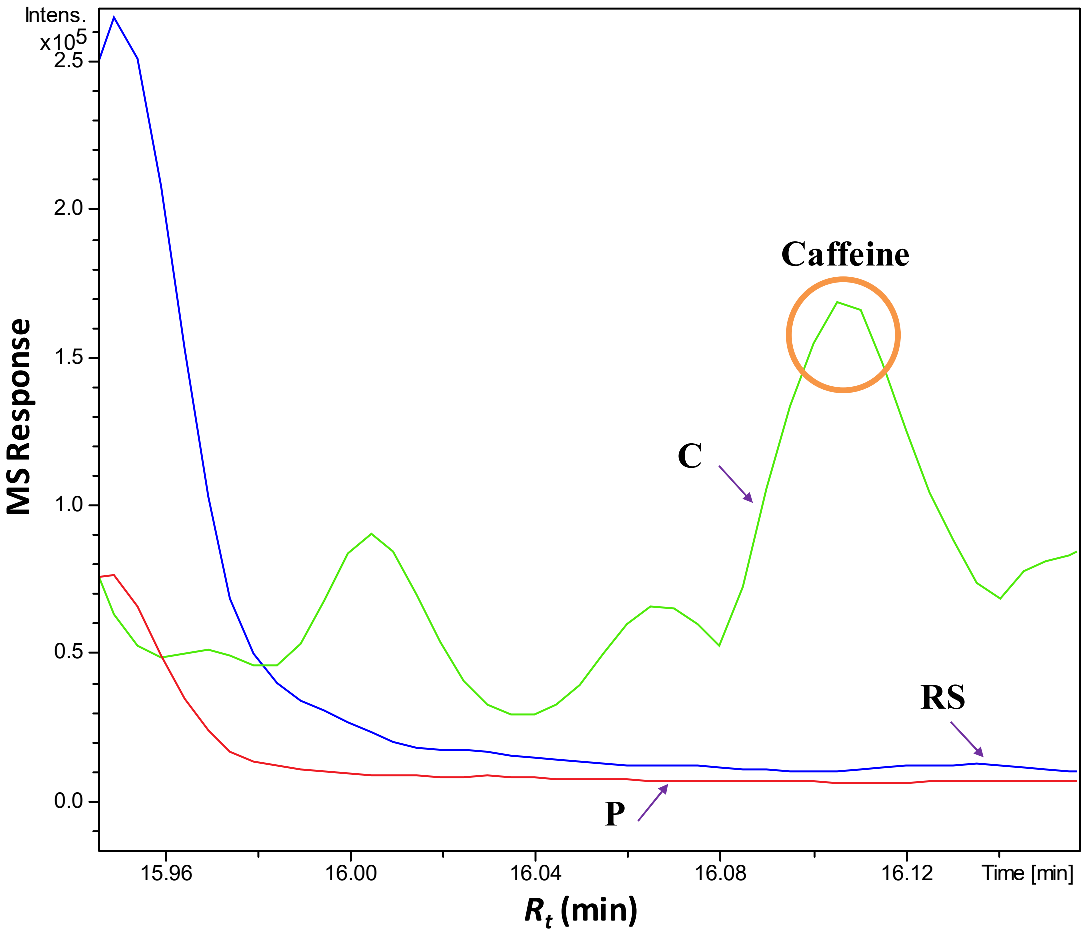

Figure 2. Representative GCMS-post silylation overlaid chromatograms (R_t 15.9-16.2 min) showing the absence of caffeine in roasted pit (RS) and pit product (P) samples, (caffeine, peak (S27), is detected at R_t 16.10 min).

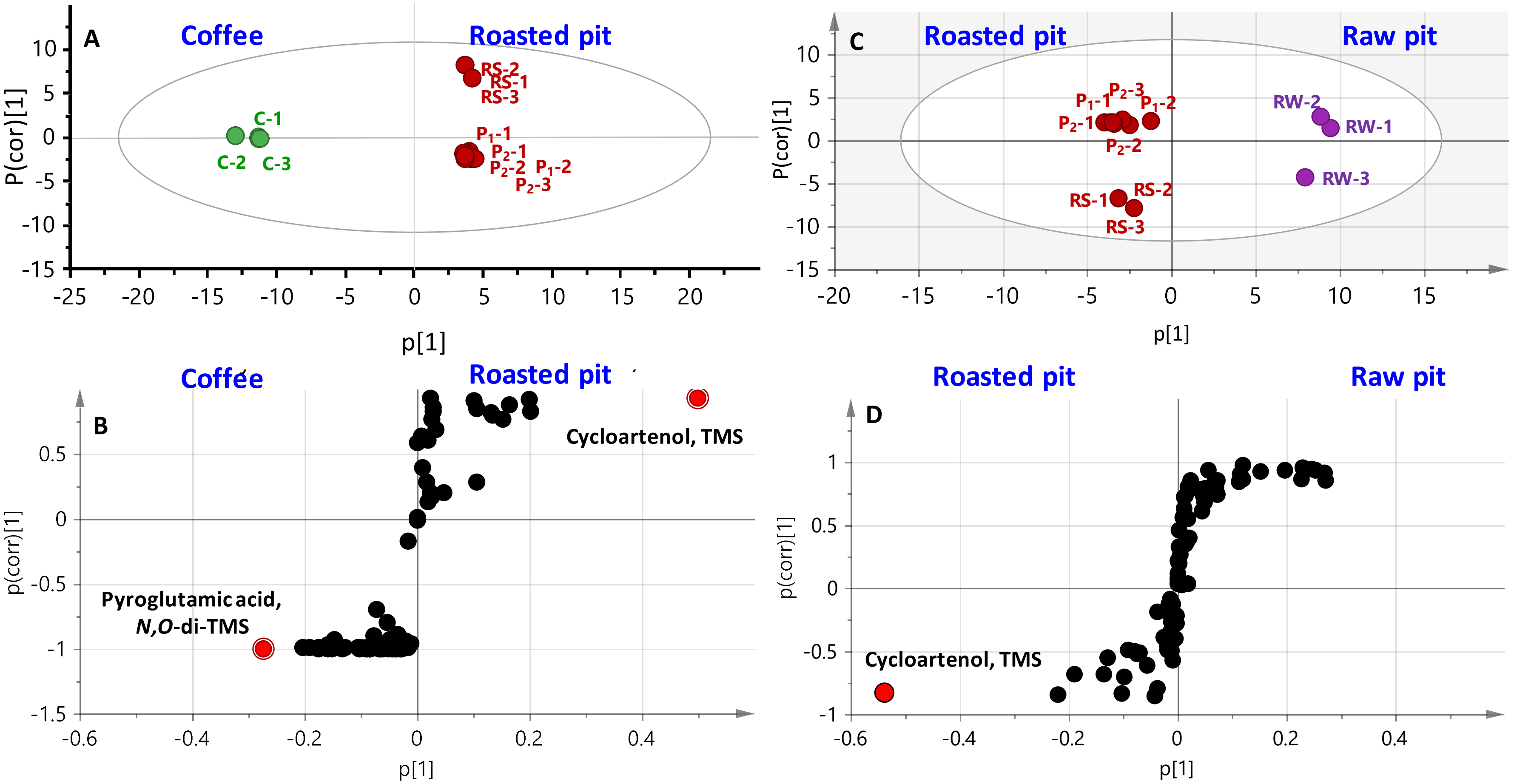

Figure 3. A. OPLS-DA score plot and **B.** loading S-plots derived from coffee samples ( ) modelled against roasted pit samples (RS, P_1, and P_2) ( ) analysed by GCMS-post silylation (*n* =3). **C.** OPLS-DA score plot and **D.** loading S-plots derived from raw pit samples ( ) modelled against roasted pit samples (RS, P_1, and P_2) ( ) analysed by GCMS-post silylation (*n* =3). The S-plot shows the covariance p[1] against the correlation p(cor)[1] of the variables of the discriminating component of the OPLS-DA model. Cut-off values of *P*<0.05 were used; variables selected are highlighted in the S-plot and identifications are discussed in the text.

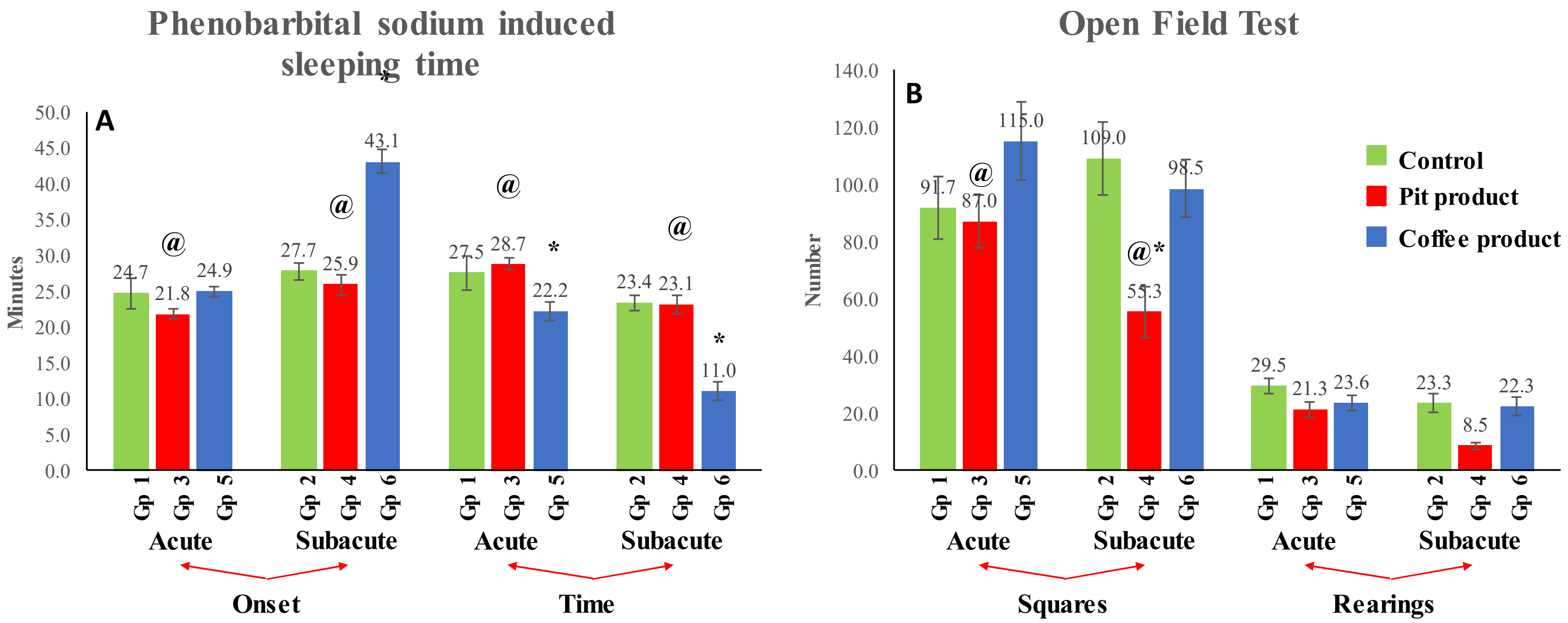

Figure 4. A. Effect of date pit product (P1) and coffee product (C) extracts on the onset and duration of sleep time during phenobarbital sodium induced sleeping test. B. Effect of date pit product (P1) and coffee product (C) extracts on the onset and duration of sleep time during phenobarbital sodium induced sleeping test. Each bar represents mean ± S.E. (*n* =10); *Significant from control, @ significant from reference coffee extract ($P \leq 0.05$, two-way ANOVA followed by Bonferroni post hoc test).

Table 1. Effect of date pit product (P) and coffee product (C) extracts on the onset and duration of sleep time during phenobarbital sodium induced sleeping test. Each bar represents mean ± S.E. (n = 6); *Significant from control, @ significant from reference coffee extract ($P \leq 0.05$, two-way ANOVA followed by Bonferroni post hoc test).

Group	Onset time (min)	Sleeping time (min)
**1**	24.714±2.133	27.543±2.387
**2**	27.741±1.257	23.4±1.028
**3**	21.754±0.639@	28.688±0.833@
**4**	25.91±1.43@	23.126±1.27@
**5**	24.93±0.744	22.152±1.298*
**6**	43.057±1.553*	10.983±1.358*

Table 2. Effect of date pit product (P) and coffee product (C) on the number of squares crossed by mice and number of rearing during Open Field Test. Each bar represents mean ± S.E. (n = 6); *Significant from control, @ significant from reference coffee extract ($P \leq 0.05$, two-way ANOVA followed by Bonferroni post hoc test).

Group	Number of squares crossed	Number of rearings
**1**	91.66±11.039	29.50±2.940
**2**	109.0±12.816	23.33±3.218
**3**	87.00±9.19@	21.33±2.260
**4**	55.33±8.864@*	8.5±1.202
**5**	115.0±13.51	23.6± 2.870
**6**	98.5± 10.233690	22.25±3.201
